# Intravascular Coronary Imaging

**DOI:** 10.1016/j.jscai.2024.102399

**Published:** 2024-12-19

**Authors:** Jennifer Rymer, J. Dawn Abbott, Ziad A. Ali, Mir B. Basir, Denise Busman, George D. Dangas, Daniel M. Kolansky, Srihari S. Naidu, Robert F. Riley, Arnold H. Seto, Binita Shah, Evan Shlofmitz, SCAI 2024 Think Tank Coronary Consortium, Connie S. Baumgard, Rafa Cavalcante, Casey Culbertson, Crista Gaalswyk, Rob J. Miltner, Jeremy Moretz, Jeannie Niebuhr, Ann Ollivier, Krish Ramakrishnan, Bradley Serwer, Nick E.J. West, Steve Zizzo

**Affiliations:** a Duke University Hospital, Durham, North Carolina; b Lifespan Cardiovascular Institute, The Warren Alpert Medical School of Brown University, Providence, Rhode Island; c St. Francis Hospital & Heart Center, Roslyn, New York; d Henry Ford Health System, Detroit, Michigan; e MedAxiom, Jacksonville Beach, Florida; f Mount Sinai Hospital, New York, New York; g Hospital of the University of Pennsylvania, Philadelphia, Pennsylvania; h Westchester Medical Center, Valhalla, New York; i Overlake Medical Center, Bellevue, Washington; j Long Beach VA Health Care System, Long Beach, California; k NYU Grossman School of Medicine, New York, New York; l Abbott, Santa Clara, California; m Boston Scientific, Minneapolis, Minnesota; n GE Healthcare, Chicago, Illinois; o Shockwave Medical, Santa Clara, California; p Abiomed, Danvers, Massachusetts; q VitalSolution, Cincinnati, Ohio; r Philips Healthcare, San Diego, California

**Keywords:** intravascular imaging, intravascular ultrasound, optical coherence tomography, quality improvement, think tank

## Introduction

Each year at the Society for Cardiovascular Angiography & Interventions (SCAI) Annual Scientific Sessions meeting, collaborative think tanks involving interventional cardiologists, administrative partners, and members of industry are convened for each SCAI clinical practice area to discuss topics of particular interest to the group. This document presents the proceedings of the 2024 coronary session, which focused on intravascular coronary imaging (ICI). The goal of this discussion was to identify needs and promote actions by the participants, leading to a positive impact on patient care.

## Intravascular coronary imaging

Current guidelines give a class IIa recommendation for the use of intravascular ultrasound (IVUS) or optical coherence tomography to assist with the diagnosis and treatment of coronary artery disease with percutaneous coronary interventions (PCI).^1^^,^^2^ ICI offers the interventional operator significant information to guide optimal and precise PCI including an assessment of the extent of lesion and vessel calcification, accurate measurement of reference vessel diameter and lesion length, and an assessment of optimal stent expansion and procedural quality. Such measurements aid in appropriate vessel preparation and stent selection and decrease the risk of both acute and late complications including in-stent restenosis and thrombosis.^3^ There is a growing evidence base supporting the routine use of ICI for PCI. A recently published meta-analysis pooled 22 studies that randomly assigned patients to ICI vs angiographic guidance alone and demonstrated a reduction in death as well as major adverse cardiac events.^3^

**Figure 1:**
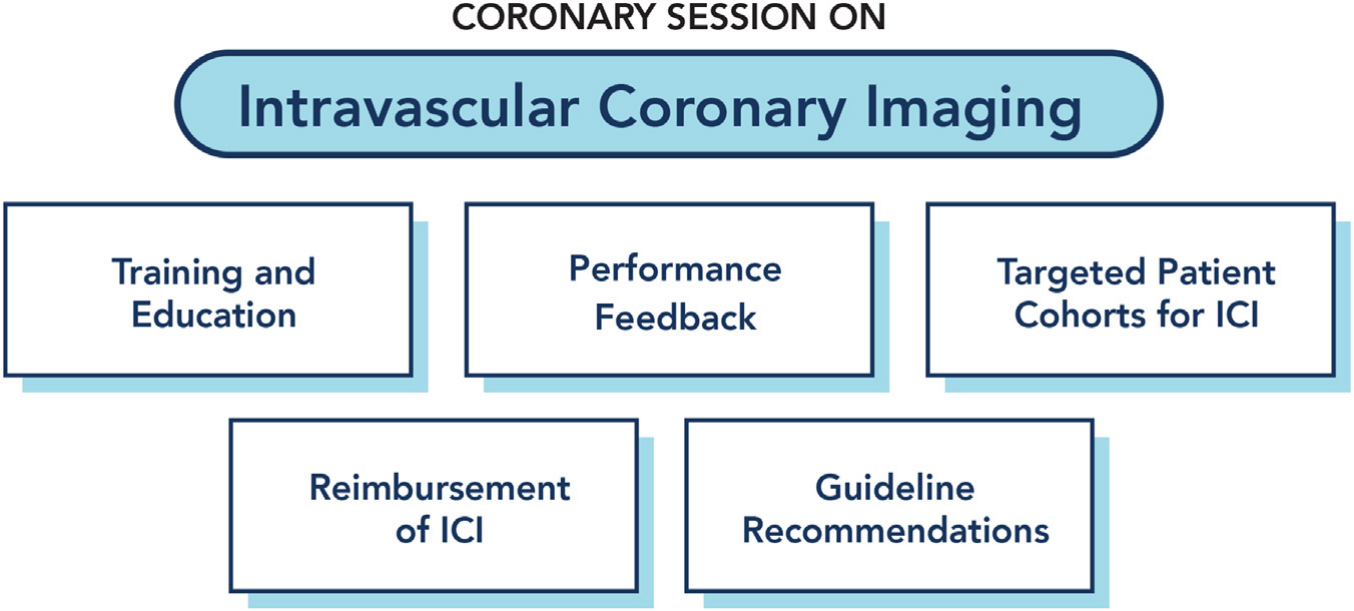
**An overview of the 2024 SCAI Think Tank proceedings for intravascular coronary imaging.** The discussions focused on 5 critical themes to shape future strategies and drive impactful changes.

Despite this evidence base, ICI remains underused in the United States. Madder et al^4^ recently published a report from the Michigan-based BMC2 Registry that showed that ICI was used in just over 16% of the PCIs included in the registry. Aside from left main PCI, the strongest predictors of ICI use were the operator that performed the PCI and the hospital where the PCI was performed, indicating that logistics and culture rather than clinical or procedural variables mostly guide use. Moreover, recent data have shown that there is significant variation in the use of IVUS across, and within, institutions with most hospitals using IVUS in less than 10% of their PCIs, and optical coherence tomography in even fewer. Our collaborative SCAI Coronary Think Tank defined 5 issues and barriers to address in order to improve the uptake of ICI across the United States: (1) training and education; (2) performance feedback to the clinical team; (3) definition of key cohorts for ICI use; (4) reimbursement; and (5) societal and regulatory support for ICI (Figure 1). These issues are addressed as follows in this report.

## Training and education

Uptake of ICI across the country is likely hindered by limited training on the setup, use and interpretation of ICI. The 2023 SCAI/American College of Cardiology/American Heart Association Advanced Training Statement on Interventional Cardiology recommends a minimum of 25 ICI procedures to be completed by the trainee along with assessment of ICI competency by the Program Director.^5^ While this recommendation may seem low for an independent operator to interpret ICI images, it is based on the current ICI use around the United States (ie, 10% of the required 250 PCI procedures for trainees).

Efforts have been made to target the training of physicians on use and interpretation of ICI through industry sponsored courses, the SCAI Scientific Sessions annual meeting, other national and international conferences and fellows’ courses. Such ongoing training is critical for interventional cardiologists to maintain and improve skills and is consistent with current medical education goals to focus on life-long learning. In addition, we propose a focus on the development of “catheterization laboratory imaging champions” who may be nonphysician clinicians, such as technicians, nurses, or advanced practice providers. These superusers would be experienced staff within the laboratory, who would optimize laboratory efficiency and quality by overseeing ICI equipment setup and image analysis that can be provided to physicians for interpretation. Such trained dedicated ICI staff champions may be particularly useful in catheterization laboratories that do not have integrated systems for ICI but rather rely on mobile carts/stations. Such imaging superusers would also provide ongoing training and support for nurses and technicians around equipment setup, image storage, and general use. This will require additional educational opportunities for these nonphysician clinicians who may champion imaging uptake.

Innovative strategies to improve the ease of use of ICI are also necessary to mitigate potential operator barriers and to help facilitate integration into routine practice. One potential path to increase ICI use by current low-volume users may be incorporation of artificial intelligence–enabled algorithms to assist in image interpretation and PCI guidance. Automated analytical packages already provide important information to assist PCI planning and optimization, including diameter/area estimations and identification of poststent expansion and malapposition. Artificial intelligence deep learning techniques to characterize lesion morphology (ie, calcium identification and quantification) are already available for ICI and may further enhance user experience, leading to a further increase in ICI uptake by current low-volume users, with potential for more prescriptive guidance subject to regulatory acceptance. An emerging alternative complementary technology may lie in computed tomography guidance, showing early promise in plaque characterization and pre-PCI planning, which may also enhance catheterization laboratory efficiency through ability to predict case complexity and need for adjunctive PCI tools in advance.

## Performance feedback

The use of intracoronary imaging during PCI may also be improved through robust performance feedback mechanisms. Such mechanisms include feedback in the form of analytical guidance or complementary technologies that assist the operator with PCI planning and optimization, as well as regular measurement and dissemination of data on individual and facility ICI use. When possible, cardiac catheterization laboratories should monitor use of ICI as a quality metric and provide reports at regular meetings. Using such feedback strategies helps overcome potential knowledge deficiencies or hesitations that may be hindering wider adoption when coupled with a culture of continuous learning in the clinical environment. Furthermore, performance metrics can be used to quantify the impact of increased imaging on patient outcomes.

## Targeted patient cohorts for ICI

The SCAI Coronary Think Tank recognizes that a goal of 100% utilization of ICI may neither be feasible nor appropriate but believes that efforts should be made to identify through guideline or consensus documents the patient cohorts in whom ICI should be used routinely. Targeted patient cohorts for near universal ICI may include patients undergoing PCI of the left mainstem, with bifurcation lesions, calcified lesions, diffuse disease, in-stent restenosis, or stent thrombosis, as well as those with high bleeding risk. An updated guidance or consensus document from professional societies outlining targeted patient populations for whom ICI is recommended in most cases may be warranted.

## Reimbursement of ICI

The SCAI Coronary Think Tank examined issues around reimbursement of ICI in PCI, and how this may be a barrier for uptake. A previous analysis of the cost-effectiveness of IVUS-guided PCI demonstrated that it is a cost-effective approach, although the time horizon to realize this benefit was around 7 years.^6^ Cost effectiveness was primarily driven by improved long-term outcomes and a reduction in associated costs, such as excessive stents at the index procedure. While this time horizon may be significant for a public payer or a closed system, such as the Veterans Health Administration, it may be more challenging for the private sector where frequent changing of health insurance carriers limits the benefit of long-term savings versus short-term costs.

Despite these issues, there are several key considerations that may be important for awareness for both individual operators and associated health systems. Currently, there is reimbursement to the operator for ICI for both the initial vessel and for the subsequent vessel. An educational campaign around ICI can help to raise awareness among interventional cardiologists, not only of the clinical benefits of imaging but also of the current reimbursement for ICI. In complex cases, as highlighted in the previous section, ICI use would be cost-favorable for the health system and/or practice.

## Guideline recommendations

The SCAI Coronary Think Tank recognizes that a goal of increased ICI uptake may be best accomplished with an update in guidelines that upgrade the class of recommendation for ICI use, particularly in complex anatomical cohorts. Given the growing body of evidence demonstrating clinical benefit of ICI in reducing stent thrombosis, target lesion and vessel revascularization, and all-cause mortality, consideration should be given for a class I recommendation for consideration of ICI in subsets where the benefits are particularly robust. Expert consensus or guidance documents in the interim from SCAI may aid in underscoring the need for such an update in guideline recommendations. Ultimately, such an update would likely be most effective in changing incentives and behaviors from payers, health systems, and operators around ICI use.

## Conclusion

The 2024 SCAI Coronary Think Tank convened to discuss current barriers and potential mitigating strategies for these barriers around the limited uptake of ICI in the United States. Such barriers include operator experience in the interpretation of ICI images, lack of nonphysician ICI champions or superusers in the catheterization laboratory, lack of emphasis or consensus on the defined patient cohorts for which ICI should generally be used, reimbursement issues for the health system and hospital, and a lack of a class I guideline recommendation for ICI for any defined subset. Our group identified various strategies to overcome these barriers, as outlined in this proceedings document. Overall, a focus on developing nonphysician catheterization laboratory champions who can become pillars of change within the local environment and a focus on encouraging ICI use in certain higher risk PCI procedures through professional consensus and guideline documents, coupled with continuing life-long physician education and appropriate reimbursement, will be most effective in increasing ICI uptake around the country.
